# Spleen Stiffness for Predicting Varices Needing Treatment: Comparison between Two Different Elastography Techniques (Point vs. 2D-SWE)

**DOI:** 10.1155/2021/6622726

**Published:** 2021-03-28

**Authors:** Renata Fofiu, Felix Bende, Raluca Lupuşoru, Roxana Şirli, Alina Popescu, Ioan Sporea

**Affiliations:** Department of Gastroenterology and Hepatology, Victor Babeş University of Medicine and Pharmacy, Timişoara, Romania

## Abstract

The study aimed to establish the benefits of using spleen stiffness values measured by two elastography techniques as noninvasive markers for predicting varices needing treatment and comparing their performances. A prospective study was performed, including 107 subjects with compensated liver cirrhosis, who underwent upper digestive endoscopy, as well as spleen stiffness measurements by means of two elastography techniques: pSWE (point shear wave elastography using Virtual Touch Quantification-Siemens Acuson S2000) and 2D-SWE (2D-shear wave elastography-LOGIQ E9, General Electric). Reliable spleen stiffness measurements were obtained in 96.2% (103/107) patients by means of 2D-SWE and in 94.4% (101/107) subjects with pSWE; therefore, 98 subjects were included in the final analysis, of which 40.8% (40/98) had varices needing treatment. The optimal spleen stiffness cut-off value by 2D-SWE for predicting varices needing treatment was 13.2 kPa (AUROC 0.84), while for pSWE, it was 2.91 m/s (AUROC 0.90). Based on AUROC comparison, no difference between the performance of the two techniques for predicting varices needing treatment was found (*p*=0.1606). In conclusion, spleen stiffness measured by either 2D-SWE or pSWE is a reliable surrogate marker, with good feasibility, applicability, and predictive accuracy for varices needing treatment, with no significant difference between techniques.

## 1. Introduction

Portal hypertension (PH) is a frequent complication of liver cirrhosis, leading to the development of esophageal varices (EV), one of the most serious complications related to PH. The gold standard method to assess PH is the measurement of the hepatic vein pressure gradient (HVPG) [1].

Besides being an invasive method, HVPG is costly, is not widely available, and requires expertise. Upper digestive endoscopy is the gold standard method for diagnosing esophageal and gastric varices. Nevertheless, a fairly high percentage of cirrhotic patients do not develop varices needing treatment (VNT), making endoscopy a nonideal screening test, due to its invasive characteristic, high costs, and the associated patient discomfort [2].

Therefore, the introduction of noninvasive markers able to predict the stage of PH (clinically significant when HVPG is >10 mmHg or not clinically significant) could help define the most opportune moment to perform endoscopy or other invasive techniques and also identify patients that need to be directed to HVPG measurements.

In recent years, the arsenal of noninvasive methods available for the evaluation of PH has increased. Besides serum markers, which do not have an optimal correlation with PH [3] and certain ultrasound signs of PH, which are sometimes difficult to visualize in early stages, ultrasound-based elastography is an increasingly used method for this purpose [4–6].

Liver stiffness (LS) is one of the most widely studied and validated noninvasive predictors of clinically significant portal hypertension (CSPH) and VNT. LS using Transient Elastography (TE) is an easily reproducible, noninvasive method that has been extensively studied and was found to have a good correlation with HVPG and the presence of EV [7–9]. A very good correlation between LS measurements (LSM) performed by 2D-shear wave elastography (2D-SWE) techniques and the presence of CSPH was also demonstrated in published studies [10–15].

Although LS is a valuable noninvasive marker for predicting PH, in situations when LSM are difficult to perform (lack of acoustic window, multifocal hepatocellular carcinoma, intrahepatic metastasis, and biliary obstruction), spleen elastography is a reliable option.

In the last years, various studies have focused on evaluating spleen stiffness measurement (SSM) and its correlation with PH. Splenomegaly is an important clinical sign used for the diagnosis of liver cirrhosis. The possible relationships between splenomegaly and portal hypertension have been intensely debated and it was concluded that splanchnic congestion and/or hyperplasia and fibrosis of the splenic tissue are the most important factors leading to splenomegaly. These aspects prove that besides enlargement, the spleen also reacts by changing its density, which is a physical characteristic that can be assessed using elastography [16]. Initially, studies demonstrated a definite and reproducible correlation between SSM by TE and the presence of PH [16]. Later on, similar results were also found with point shear wave elastography (pSWE) and 2D-SWE [12, 13, 17, 18]. Despite well-defined evidence that spleen stiffness is a reliable noninvasive marker for predicting PH, the elastography technique's choice remains controversial.

The aim of this study was to establish the performance of SS using two elastography techniques: point shear wave elastography (pSWE) and 2D-shear wave elastography (2D-SWE) as a noninvasive marker for predicting VNT, in patients with compensated liver cirrhosis and to compare the performances of the two techniques.

## 2. Materials and Methods

### 2.1. Subjects

The study prospectively included 107 subjects that were previously diagnosed with compensated liver cirrhosis of mixed etiologies.

Inclusion criteria for all the subjects were the ability to provide informed consent, age ≥18 years, previous diagnosis of compensated liver cirrhosis based on clinical, biological, and elastography (LS by TE >12.5 kPa) criteria [19].

Exclusion criteria were LS by TE ≤12.5 kPa, patients with ascites, aminotransferases higher than three times the upper normal limit, patients with signs of biliary obstruction, liver congestion secondary to heart failure, patients with focal liver lesions or portal vein thrombosis, patients on nonselective beta-blockers treatment, and patients with noncirrhotic PH.

All subjects included in the study underwent both upper digestive endoscopy and SSM using two elastography techniques: pSWE-using Virtual Touch Quantification (VTQ) technology (Acuson S2000-Siemens Medical Solutions) and 2D-SWE (LOGIQ E9-General Electric), generally during the same admission period, but not at more than one-month interval. LS evaluation was also performed using both TE (FibroScan; EchoSens, Paris, France) and 2D-SWE (LOGIQ E9-General Electric) as previously described [14, 20, 21].

The following data were collected: age, gender, body mass index (BMI), etiology of liver cirrhosis, spleen size, SS values with both techniques, LS values by TE, and 2D-SWE (LOGIQ E9-General Electric). Each elastography evaluation was preceded by an abdominal ultrasound examination, during which the spleen size—diameter of the longitudinal axis—was measured. All patients with alcohol-related liver cirrhosis included in our study stated they were abstinent from alcohol consumption.

All the participants signed informed consent for performing the elastography measurements. The study was approved by the Ethics Committee and was performed complying with the last revised version of the Helsinki Declaration.

### 2.2. Elastography Evaluation

SSM and LSM were performed by a single experienced operator, complying with the recommendations of the latest guidelines [22, 23]: with the patient in fasting conditions (at least 6 h), in a supine position, with the arm in maximum abduction, by intercostal approach, in the superior pole of the spleen and by avoiding the spleen capsule for spleen stiffness, and in the right liver lobe for liver stiffness.

### 2.3. Point Shear Wave Elastography Technique

SSM by p-SWE were performed using the Siemens Acuson S2000 ultrasound system with the Virtual Touch™ Tissue Quantification software. Using a convex array probe of 1–6 MHz, a region of interest (ROI) (with preset dimensions 1/0.5 cm box and the maximum evaluable depth of 5.5 cm) was placed in a homogeneous area of the splenic parenchyma, at the level of the superior pole of the spleen. Afterward, the patient was asked to suspend breathing and the measurement was initiated. In each patient, ten valid consecutive measurements were performed and the median value was calculated, the results being expressed in meters/second (m/s). Only the measurements with IQR (interquartile range interval = difference between the 75th and the 25th percentile, essentially the range of middle 50% of the data) <0.30 were considered reliable. Measurements were considered failures when no value was obtained after 10 attempts.

### 2.4. 2D Shear Wave Elastography Technique

SSM and LSM by 2D-SWE were performed using a LOGIQ E9 system (GE Healthcare, Wauwatosa, WI, USA) (version 2.0), using the C1-6-D convex probe. The SWE ROI was placed in a well-visualized area of the splenic parenchyma, at the level of the superior pole of the spleen. In some cases (spleen size <12 cm) when a homogeneous load of the ROI was not obtained, the measurement was performed closer to the centre of the spleen. For LS, the ROI was placed at least 1–2 cm below the liver capsule, in an area free of large vessels. While the patient was asked to suspend breathing, image acquisition was initiated. The system records several second loops and then the measurements are performed frame by frame. A circular measurement ROI is placed in each frame and the measurement is obtained. A single measurement was performed in each loop and ten consecutive measurements are acquired for each subject. The average stiffness, expressed in terms of Young's Modulus within each measurement region, was automatically recorded by the system in a worksheet. The system consequently calculates the median value and IQR of the valid measurements. Reliable SSM/LSM were defined as the median value of 10 measurements acquired in a homogenous area, with an IQR/M <0.30. Measurements were considered failures when no value was obtained after 10 attempts.

### 2.5. Upper Digestive Endoscopy

All subjects included in the study underwent upper digestive endoscopy performed by an experienced clinician, blinded to the spleen and liver stiffness measurements, usually during the same admission time. In cases where upper digestive endoscopy was performed during different admissions, the procedure was performed not later than one month. The presence and the grade of EV, as well as the presence of gastric varices or portal hypertensive gastropathy, were recorded. All varices that were described as grade 1 without red wale marks were defined as varices not needing treatment. Consequently, grade 1 with red wale marks or medium/large (grade 2/3) was defined as VNT. Any types of gastric varices were defined as VNT [1, 24].

### 2.6. Statistical Analysis

The statistical analysis was performed using MedCalc software, version 12.5.0.0 (MedCalc program, Belgium), and SPSS, version 17.0 (IBM Statistics). The Kolmogorov–Smirnov test was used for testing the distribution of numerical variables.

Numerical variables which have normal distribution are presented as means ± standard deviation, while in cases of variables with nonnormal distribution, median values and range intervals were used. Categorical variables were presented as the number or proportion of subjects with or without the specific characteristic.

Student's *t*-test was used for assessing the differences between groups for continuous variables with a normal distribution and the nonparametric test; Mann–Whitney *U* test was used for variables with nonnormal distribution. Group comparisons of categorical variables were performed using Pearson's *χ*^2^-test.

Areas under receiver operating characteristic curves (AUROC) were calculated for 2D-SWE and pSWE values in order to identify discriminating cut-offs for SS. The optimal cut-off values were calculated from AUROC curve analysis, by using the Bayesian analysis, using the optimal criterion (the cut-off value with the highest sum of Se and Sp), and avoiding the misclassification of true positives subjects. Rule-out and rule-in cut-off values were determined from the AUROC curve analysis. Cut-off values that optimized specificity and sensitivity, respectively, were chosen. Positive predictive value (PPV defined as the ratio between the true positive cases and all the positive cases), negative predictive value (NPV defined as the ratio between the true negative cases and all the negative cases), and diagnostic accuracy (defined as the ratio between the sum of true positive and true negative cases and the total number of cases) were calculated. 95% confidence intervals (CI) were determined for each predictive test and a *p* value below 0.05 was considered to concede statistical significance. The 3 × 2 tables with an intention to diagnose approach were used to assess the clinical performance of our methods and to avoid the overestimation of diagnostic accuracy by excluding the nonevaluable subjects. The nonevaluable results were considered either positive or negative in comparison with the standard method (in our case, upper digestive endoscopy); if considered positive, they were added to the number of false-negative subjects, and if considered negative, they were added to the number of false-positive subjects.

## 3. Results

### 3.1. Baseline Characteristics

SSM were assessed by means of pSWE and 2D-SWE in 107 patients. Reliable SSM were obtained in 96.2% (103/107) of patients by means of 2D-SWE and in 94.4% (101/107) od subjects by means of pSWE (*p*=0.76). Subjects were divided according to spleen size into two groups: the first group included subjects with normal spleen size (≤12 cm, *n* = 28) and the second included subjects with an enlarged spleen (>12 cm, *n* = 79). The proportion of unreliable SSM by 2D-SWE was significantly higher in patients with the normal spleen (*p*=0.0225), while for pSWE, no statistically significant differences were found between the proportions of unreliable SSM in the two groups (*p*=0.2117).

BMI (kg/m^2^) mean values were significantly higher for patients with unreliable SSM as compared to those with reliable SSM (30.2 ± 3.4 kg/m^2^ versus 24.5 ± 2.9 kg/m^2^; *p* < 0.0001).

**Figure 1:**
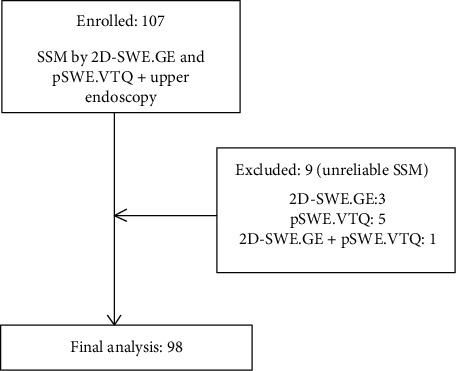
Characteristics of subjects enrolled in the study.

After excluding patients in whom valid measurements were impossible to obtain by either of the elastography techniques, 98 subjects were included in the final analysis (Figure 1).

60.8% (45/74) of the subjects with viral hepatitis had SVR (HCV patients) or were undergoing oral antiviral treatment (HBV patients). Mean SS values were significantly lower for patients with SVR (sustained virologic response) or undergoing chronic antiviral treatment compared to those without (12.06 ± 1.72 kPa vs. 17.9 ± 2.6 kPa, *p* < 0.0001 for 2D-SWE.GE and 2.78 ± 0.32 m/s vs. 3.32 ± 0.56 m/s, *p* < 0.0001 for pSWE.VTQ, resp.). The subjects' characteristics are summarized in Table 1.

**Figure 2:**
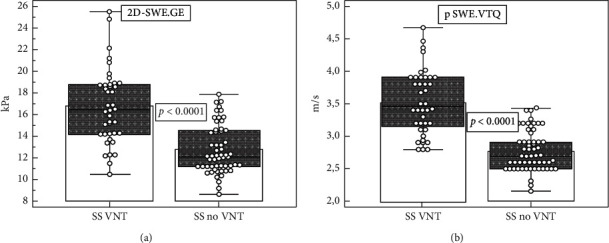
Comparison of mean SS by 2D-SWE.GE and pSWE.VTQ values in patients with VNT versus patients with no VNT.

Mean SSM values were significantly higher for patients with VNT as compared to those without, for both techniques (16.74 ± 3.42 kPa vs. 12.71 ± 2.2 kPa, *p* < 0.0001 for 2D-SWE; 3.52 ± 0.49 m/s vs. 2.7 ± 0.3 m/s, *p* < 0.0001 for pSWE) (Figure 2).

### 3.2. Performance of SSM for Predicting VNT

The optimal SSM cut-off values for both 2D-SWE.GE and pSWE.VTQ for predicting VNT, as well as the rule-out and rule-in cut-off values, are summarized in Table 2.

The optimal cut-off values for LS by 2D-SWE.GE and spleen size for predicting VNT were 12.1 kPa (AUROC 0.86, Se 85%, Sp 69%, PPV 65.4%, and NPV 87%) and 12.9 cm (AUROC 0.72, Se 85%, Sp 50%, PPV 54%, and NPV 82.9%), respectively.

**Figure 3:**
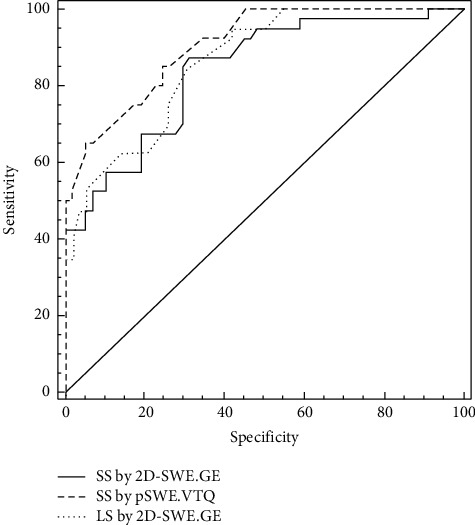
Comparison between receiver operating characteristics for SS (2D-SWE and pSWE) and LS (2D-SWE).

Based on AUROC comparison (AUC 0.84 vs. AUC 0.90), no difference between the performance of SS assessed with the two techniques for predicting VNT (*p*=0.16) was found, nor between LS by 2D-SWE.GE (AUC 0.86) and SS, regardless of the technique (AUC 0.86 vs. AUC 0.84, *p*=0.57 for 2D-SWE; AUC 0.86 vs. AUC 0.90, *p*=0.3 for pSWE) (Figure 3).

The proportion of subjects correctly classified after applying the previously established cut-off values is summarized in Table 3.

### 3.3. Performance of Baveno VI Criteria Combined with SSM for Predicting VNT

Baveno VI criteria (LS <20 kPa and platelet count >150 × 10^9^ cells/L) were applied, 20/98 subjects were within Baveno VI criteria, and 1/20 (5%) subjects had VNT. When the Expanded-Baveno VI criteria were applied (LS <25 kPa and platelet count >110 × 10^9^ cells/L), 39/98 patients were within criteria and 3/39 (7.7%) had VNT.

When the SSM by 2D-SWE.GE cut-off value was used, 45/98 (46%) subjects had SSM <13.2 and 3/45 (6.6%) of them had VNT. Using SSM cut-off values by pSWE.VTQ, 49/98 (50%) subjects had SSM <2.9 m/s and 4/49 (6.1%) had VNT.

Consequently, when both SSM by 2D-SWE.GE <13.2 kPa and Baveno VI criteria were applied, 16/98 (16.3%) subjects were within criteria and none of them had VNT. When the Expanded-Baveno VI criteria were used instead, 26/98 (26.5%) subjects were within criteria and none of them had VNT. Baveno VI criteria were also tested with SS by pSWE.VTQ <2.9 kPa, 17/98 (17.3%) subjects were within criteria and none of them had VNT. When the Expanded-Baveno VI criteria were used instead, 25/98 (25.5%) subjects were within criteria and none of them had VNT.

The percentage of patients who can safely avoid upper digestive endoscopy was 16.3% for SS by 2D-SWE.GE combined with Baveno VI criteria and 26.5% when combined with Expanded-Baveno VI criteria (*p*=0.11). When SS by pSWE.VTQ was used together with Baveno VI criteria, 17.3% of the upper digestive endoscopies could have been avoided, while when used together with Expanded-Baveno VI, 25.5% of patients could safely avoid upper digestive endoscopy (*p*=0.22).

## 4. Discussions

The development of CSPH is a major step in the natural history of patients with advanced chronic liver disease (ACLD). For risk and prognosis assessment, it is crucial to evaluate in all patients, at the time of diagnosis, the status of PH.

Because HVPG and upper digestive endoscopy are not widely available and are unpleasant for patients, easily reproducible, surrogate markers are needed. So far, liver elastography proved to be a very good prognostic marker for the presence of CSPH and VNT [7–10, 14], but considering the fact that there are many situations in clinical practice when LSM are impossible to perform, SSM represents a reliable alternative. Although data regarding the superiority of SSM compared to LSM for predicting CSPH are inconsistent among studies, a meta-analysis that evaluated 16 studies concluded that SSM are superior to LSM [25].

Previous studies have already highlighted the usefulness of SSM as a noninvasive marker for predicting CSPH, the majority of them being performed using Transient Elastography (TE) [16, 26–28]. Despite this well-defined evidence, SSM by TE have not been used routinely since it has several technical limitations: low applicability and reproducibility in normal-sized spleen and ceiling effect at 75 kPa, impairing risk stratification of patients [2]. Recently, a novel FibroScan software dedicated to spleen stiffness evaluation has managed to overcome some of the disadvantages of the standard FibroScan, even so, pSWE and 2D-SWE techniques still have the advantage of allowing direct visualization of the splenic parenchyma. Therefore evaluating the performance of other elastography techniques is justified [29]. The present study evaluates the accuracy of SSM for predicting the presence of VNT using two different elastography techniques: pSWE and 2D-SWE. Similar rates of reliable SSM by both techniques (96.2% vs. 94.4%; *p*=0.76) were found. Previous studies confirmed the good feasibility of SSM by pSWE; Takuma et al [18] obtained reliable SSM in 95.5% of the subjects, while Bota et al. [30] obtained them in 95.2% of the subjects, using the same technique. The good feasibility (90.2%) of SSM using a 2D-SWE technique implemented on a different ultrasound system (Aixplorer, Supersonic Imagine) was also confirmed by a published study [31].

After analysing the causes that led to SSM failure in our study, we found out that 78% (7/9) of the subjects in whom no reliable measurements could be obtained had normal spleen size (≤12 cm). In only one patient (1/9), we were unable to obtain reliable measurements by any of the two techniques used. This aspect is important for clinical practice. When one of the methods is not feasible, the other may be used instead. Regarding BMI, higher BMI was associated with the impossibility to obtain reliable SSM by either of the techniques. Previous studies have shown that normal spleen size and high BMI are the most frequent causes that lead to SSM failure [11, 16, 27].

As it was already shown in other studies [10, 32–34], in our group, the mean SSM values determined by both techniques were significantly higher for patients with VNT as compared to those without. The diagnostic accuracy of SSM by both p-SWE and 2D-SWE for predicting the presence of VNT was evaluated and good diagnostic accuracy was found (AUROC 0.90 and 0.84, resp.; *p*=0.1606).

Similar results were obtained by Takuma et al. [18] using pSWE (Siemens Acuson S2000). The AUROC for predicting the presence of VNT ranged between 0.92 and 0.94 depending on the etiology. A more recent study concluded that SSM by pSWE (Siemens Acuson S2000) can predict the presence of large esophageal varices with an AUROC of 0.97 [34].

Regarding SS evaluation by 2D-SWE, a recent study concluded that SSM by 2D-SWE (Aixplorer, Supersonic Imagine) is a reliable tool for ruling out the presence of VNT, with a NPV of 91.3%, offering an AUROC of 0.854, which is similar to our results [25]. Grgurević et al. evaluated the performance SSM assessed with 2D-SWE (Aixplorer, Supersonic Imagine) as noninvasive predictors of EV and showed a NPV of 86.6% to exclude EV in compensated patients [35].

An important issue that needs to be discussed is the fact that our cut-off values are on average lower compared to those already published [19, 34, 36] for both techniques. International guidelines [22, 23] stipulate that each ultrasound system has its own cut-off values for predicting various stages of fibrosis and also for predicting CSPH and the presence of EV and they have to be established for every method independently. Besides that, a high proportion of the subjects included in our study were at SVR or on chronic antiviral treatment and this could have been a cause for the lower cut-off values that we have established. Moreover, the mean SS values for patients with SVR (HCV patients) or chronic antiviral treatment (HBV patients) were significantly lower compared to the naive patients for either of the techniques (*p* < 0.0001).

Based on AUROC comparison (AUC 0.90 vs. AUC 0.84), no difference between the performance of SSM by the two techniques for predicting VNT was found in our study (*p*=0.16). A more detailed analysis revealed that there were no differences regarding the proportion of patients correctly classified as having or not having VNT, using the optimal SSM cut-offs (76.5% vs. 79.6%, *p*=0.72), the rule-out cut-offs (96% vs. 92.1%, *p*=0.92), and the rule-in cut-offs (82.6% vs. 89.3%, *p*=0.07) proposed for the two techniques (pSWE vs. 2D-SWE). Both methods performed better to rule-out than to rule-in VNT.

To avoid overestimating diagnostic accuracy by excluding subjects with unreliable measurements, we used the 3 × 2 tables with an intention to diagnose approach to assess the clinical performance of our methods. By including all subjects, the results are closer to those encountered in clinical practice and the diagnostic accuracy was 72% for pSWE.VTQ and 71% for 2D-SWE.GE, while after excluding the subjects with unreliable SSM, the diagnostic accuracy was 79% for pSWE.VTQ and 76% for 2D-SWE.GE, respectively. As expected by including the nonevaluable results, the diagnostic accuracy decreased, but the differences are not significant (*p*=0.3 and *p*=0.51, resp.); this aspect can be explained by the fact that the proportion of subjects excluded in the first place was not so high.

The good performance of these methods to evaluate SS as a predictor for VNT is encouraging, and they can be used in clinical practice, whenever SS evaluation by TE is not available or cannot be performed. Another relevant aspect highlighted in this paper is that there are no differences between the feasibility, applicability, and predictive accuracy of the two proposed methods (pSWE and 2D-SWE) for SS evaluation, so the choice of method depends only on local availability and expertise.

Studies have shown that a multiparametric or step-by-step approach has better performance in identifying EV or predicting CSPH as compared to the individual use of noninvasive markers [17, 21, 27, 37]. In addition to the well-known and validated scores, in a recent study, a simple clinical and biological score (Liaoning Score) was formulated and had a good performance for detecting EV in patients diagnosed with liver cirrhosis (AUC 0.80) [37].

Concerning the combined use of SS together with the Baveno VI and Expanded-Baveno VI criteria, our results are similar to those of other published studies; this approach leads to an increased proportion of upper digestive endoscopies that could be avoided and a reduction in the proportion of subjects with missed VNT [24, 38–41]. In fact, studies have already shown that a multiparametric or step-by-step approach has better performance in identifying EV or predicting CSPH as compared to the individual use of noninvasive markers.

The most important limitations of the study are the number of subjects included, which is not very high and the absence of a control group for validating our results, as well as the fact that the upper digestive endoscopy and not HVPG was used to stratify the severity of PH. Besides the limitations related to the cohort, several aspects should be mentioned: the study included only patients with liver cirrhosis and did not include patients with compensated advanced chronic liver disease (LS by TE between 8 and 12.5 kPa), although in this category of patients, screening with noninvasive techniques is important. In addition, given the distribution of etiology in the study cohort, our results refer mostly to patients with HCV infection, while they need confirmation in other etiologies that were less prevalent in our cohort. It is noteworthy that most patients with viral hepatitis (HCV, HBV) were patients with SVR or undergoing chronic antiviral treatment. Regarding subjects with ALD, an important aspect is that we did not use an objective quantification test for alcohol intake at the time of evaluation. The lack of SSM by TE is another limitation that should be mentioned.

## 5. Conclusions

Spleen stiffness is a reliable surrogate marker, with good feasibility, applicability, and predictive accuracy for varices needing treatment, using both 2D-SWE and pSWE, with no significant difference between techniques. None of the studied methods was superior to the other, so the choice of the method for evaluating the SS depends only on the expertise, experience, and local availability.

## Figures and Tables

**Table 1:** Main characteristics of subjects enrolled in the study.

Parameter	All subjects with reliable SSM (*n* = 98)	No VNT *n* = 58 (59.2%)	VNT *n* = 40 (40.8%)	*p* value
Age	59 ± 9.47	59.9 ± 8.72	57.2 ± 10.7	*p*=0.17

Gender				
Male	44% (43/98)	32.8% (19/58)	60% (24/40)	*p*=0.013
Female	56% (55/98)	67.2% (39/58)	40% (16/40)	*p*=0.013

Etiology				
HCV	71.5% (70/98)	77.6% (45/58)	62.5% (25/40)	*p*=0.16
HBV	4% (4/98)	5.2% (3/58)	2.5% (1/40)	*p*=0.86
ALD	12.3% (12/98)	6.9% (4/58)	20% (8/40)	*p*=0.01
NAFLD	8.2% (8/98)	5.2% (3/58)	12.5% (5/40)	*p*=0.35
PBC	4% (4/98)	5.1% (3/58)	2.5% (1/40)	*p*=0.9

Platelet count (x10^9^/L)	121.8 ± 56.9	135.6 ± 55.1	106.7 ± 61.6	*p*=0.017
LSM by 2D-SWE.GE	12.54 ± 2.27	11.5 ± 1.4	14.16 ± 2.34	*p* < 0.0001
SSM by 2D-SWE.GE<	14.36 ± 3.39	12.71 ± 2.2	16.74 ± 3.4	*p* < 0.0001
SSM by pSWE.VTQ	3.08 ± 0.54	2.77 ± 0.3	3.52 ± 0.49	*p* < 0.0001
Spleen size (cm)	13.68 ± 1.93	13.8 ± 1.67	14.55 ± 1.97	*p*=0.0452
EV 0	32/98 (32.7%)	32/58 (55.2%)	0/40 (0%)	*p* < 0.0001
EV 1	26/98 (26.5%)	26/58 (44.8%)	0/40 (0%)	*p* < 0.0001
EV 2	31/98 (31.6%)	0/58 (0%)	34/40 (85%)	*p* < 0.0001
EV 3	4/98 (4.1%)	0/58 (0%)	6/40 (15%)	*p* < 0.0001
EV + GV	5/98 (5.1%)	0/58 (0%)	5/40	*p* < 0.0001

VNT: varices needing treatment; *n*: number; HCV: hepatitis C virus; HBV: hepatitis B virus; ALD: alcoholic liver disease; NAFLD: nonalcoholic fatty liver disease; PBC: primary biliary cirrhosis; LSM: liver stiffness measurements; SSM: spleen stiffness measurements; EV: esophageal varices; and GV: gastric varices.

**Table 2:** SS optimal cut-off values and rule-out and rule-in cut-off values for predicting VNT.

Optimal cut-off values								
Parameter	Cut-off	AUC	Se (%)	Sp (%)	PPV (%)	NPV (%)	*p* value	
SS by 2D-SWE	13.2 kPa	0.84	87.5	69.0	66.0	88.9	*p*=0.16	
**SS by pSWE**	2.91 m/s	0.90	85.0	75.8	70.8	88.0		

Rule-out and rule-in cut-off values								
2D-SWE	Rule-out^*∗*^	11.4 kPa	0.84	97.5	42.0	53.4	96.0	**<0.001**
	Rule-in^*∗∗*^	16.7 kPa	0.84	47.5	93.1	86.4	72.4	**<0.001**
**pSWE**	Rule-out^*∗*^	2.80 m/s	0.90	92.5	60.4	61.7	92.1	**<0.001**
	Rule-in^*∗∗*^	3.42 m/s	0.90	50.0	98.3	95.5	75.0	**<0.001**

Se: sensitivity; Sp: specificity; PPV: positive predictive value; NPV: negative predictive value; and AUROC: area under a receiver operating curve. *∗*^∗^Cut-off values that optimized sensitivity were chosen. ^*∗∗*^Cut-off values that optimized specificity were chosen.

**Table 3:** Classification of subjects according to the optimal, rule-out, and rule-in SSM cut-off values.

	SSM (kPa)	Correctly classified (%)	*p* value	Misclassified (%)	*p* value
Optimal^*∗*^	2D-SWE	76.5	*p*=0.725	24.5	*p*=0.605
	pSWE	79.6		20.4	

Rule-out^*∗∗*^	2D-SWE	96	*p*=0.925	4.0	*p*=0.925
	pSWE	92.1		7.9	

Rule-in^*∗∗∗*^	2D-SWE	82.6	*p*=0.077	17.4	*p*=0.077
	pSWE	89.3		10.7	

SSM: spleen stiffness measurements; Se: sensitivity; Sp: specificity; PPV: positive predictive value; NPV: negative predictive value; and AUROC: area under a receiver operating curve. ^*∗*^Cut-off values with the higher sum of sensitivity and specificity were chosen. ^*∗∗*^Cut-off values that optimized sensitivity were chosen. Cut-off values that optimized specificity were chosen.

## Data Availability

The data underlying the findings of the study are available on request to the corresponding author (e-mail address: bende.felix@umft.ro).

## References

[1] Garcia-Tsao G., Abraldes J. G., Berzigotti A., Bosch J. (2017). Portal hypertensive bleeding in cirrhosis: risk stratification, diagnosis, and management: 2016 practice guidance by the American Association for the study of liver disease. *Hepatology*.

[2] Ravaioli F., Montagnani M., Lisotti A., Festi D., Mazzella G., Azzaroli F. (2018). Noninvasive assessment of portal hypertension in advanced chronic liver disease: an update. *Gastroenterology Research and Practice*.

[3] European Association for Study of Liver (2015). Asociacion Latinoamericana para el Estudio del Higado. EASL-ALEH clinical practice guidelines: non-invasive tests for evaluation of liver disease severity and prognosis. *Journal of Hepatology*.

[4] Berzigotti A., Rossi V., Tiani C. (2011). Prognostic value of a single HVPG measurement and doppler-ultrasound evaluation in patients with cirrhosis and portal hypertension. *Journal of Gastroenterology*.

[5] Berzigotti A., Piscaglia F. (2011). Ultraschall bei Pfortaderhochdruck-Teil 1. *Ultraschall in der Medizin-European Journal of Ultrasound*.

[6] Berzigotti A., Piscaglia F. (2012). Ultraschall bei Pfortaderhochdruck-Teil 2-und EFSUMB-Empfehlungen zur Durchführung und Dokumentation von Ultraschalluntersuchungen bei Pfortaderhochdruck. *Ultraschall in der Medizin -European Journal of Ultrasound*.

[7] Shi K.-Q., Fan Y.-C., Pan Z.-Z. (2013). Transient elastography: a meta-analysis of diagnostic accuracy in evaluation of portal hypertension in chronic liver disease. *Liver International*.

[8] Li T., Qu Y., Yang B., Xue Y., Wang L. (2016). Evaluation of large esophageal varices in cirrhotic patients by transient elastography: a meta-analysis. *Revista Española de Enfermedades Digestivas*.

[9] Cheng F., Cao H., Liu J. (2018). Meta-analysis of the accuracy of transient elastography in measuring liver stiffness to diagnose esophageal varices in cirrhosis. *Medicine*.

[10] Yu J. B., Xiong H., Yuan X. C., Zhou A. Y. (2019). Liver stiffness detected by shear wave elastography predicts esophageal varices in cirrhotic patients. *Ultrasound Quarterly*.

[11] Procopet B., Berzigotti A., Abraldes J. G. (2015). Real-time shear-wave elastography: applicability, reliability and accuracy for clinically significant portal hypertension. *Journal of Hepatology*.

[12] Elkrief L., Rautou P.-E., Ronot M. (2015). Prospective comparison of spleen and liver stiffness by using shear-wave and transient elastography for detection of portal hypertension in cirrhosis. *Radiology*.

[13] Kim T. Y., Jeong W. K., Sohn J. H., Kim J., Kim M. Y., Kim Y. (2015). Evaluation of portal hypertension by real-time shear wave elastography in cirrhotic patients. *Liver International*.

[14] Stefanescu H., Rusu C., Lupsor-Platon M. (2020). Liver stiffness assessed by ultrasound shear wave elastography from general electric accurately predicts clinically significant portal hypertension in patients with advanced chronic liver disease. *Ultraschall in der Medizin-European Journal of Ultrasound*.

[15] Deng H., Qi X., Zhang T., Qi X., Yoshida E. M., Guo X. (2018). Supersonic shear imaging for the diagnosis of liver fibrosis and portal hypertension in liver diseases: a meta-analysis. *Expert Review of Gastroenterology & Hepatology*.

[16] Colecchia A., Montrone L., Scaioli E. (2012). Measurement of spleen stiffness to evaluate portal hypertension and the presence of esophageal varices in patients with HCV-related cirrhosis. *Gastroenterology*.

[17] Jansen C., Bogs C., Verlinden W. (2017). Shear-wave elastography of the liver and spleen identifies clinically significant portal hypertension: a prospective multicentre study. *Liver International*.

[18] Takuma Y., Nouso K., Morimoto Y. (2013). Measurement of spleen stiffness by acoustic radiation force impulse imaging identifies cirrhotic patients with esophageal varices. *Gastroenterology*.

[19] Castera L., Forns X., Alberti A. (2008). Non-invasive evaluation of liver fibrosis using transient elastography. *Journal of Hepatology*.

[20] Ziol M., Handra-Luca A., Kettaneh A. (2005). Noninvasive assessment of liver fibrosis by measurement of stiffness in patients with chronic hepatitis C. *Hepatology*.

[21] Fofiu R., Bende F., Popescu A. (2021). Spleen and liver stiffness for predicting high-risk varices in patients with compensated liver cirrhosis. *Ultrasound in Medicine & Biology*.

[22] Ferraioli G., Filice C., Castera L. (2015). WFUMB guidelines and recommendations for clinical use of ultrasound elastography: Part 3: liver. *Ultrasound in Medicine & Biology*.

[23] Dietrich C., Bamber J., Berzigotti A. (2017). EFSUMB guidelines and Recommendations on the clinical use of liver ultrasound elastography, update 2017 (long version). *Ultraschall in der Medizin-European Journal of Ultrasound*.

[24] De Franchis R., VI Faculty B. (2015). Expanding consensus in portal hypertension. *Journal of Hepatology*.

[25] Ma X., Wang L., Wu H. (2016). Spleen stiffness is superior to liver stiffness for predicting esophageal varices in chronic liver disease: a meta-analysis. *PLoS One*.

[26] Castera L., Pinzani M., Bosch J. (2012). Non invasive evaluation of portal hypertension using transient elastography. *Journal of Hepatology*.

[27] Stefanescu H., Grigorescu M., Lupsor M., Procopet B., Maniu A., Badea R. (2011). Spleen stiffness measurement using Fibroscan for the noninvasive assessment of esophageal varices in liver cirrhosis patients. *Journal of Gastroenterology and Hepatology*.

[28] Song J., Huang J., Huang H., Liu S., Luo Y. (2018). Performance of spleen stiffness measurement in prediction of clinical significant portal hypertension: a meta-analysis. *Clinics and Research in Hepatology and Gastroenterology*.

[29] Stefanescu H., Marasco G., Calès P. (2020). A novel spleen‐dedicated stiffness measurement by fibroscan improves the screening of high‐risk oesophageal varices. *Liver International*.

[30] Bota S., Sporea I., Sirli R., Popescu A., Dănilă M. (2010). Spleen assessment by acoustic radiation force impulse elastography (ARFI) for prediction of liver cirrhosis and portal hypertension. *Medical ultrasonography*.

[31] Karagiannakis D. S., Voulgaris T., Koureta E., Chloupi E., Papatheodoridis G. V., Vlachogiannakos J. (2019). Role of spleen stiffness measurement by 2D-shear wave elastography in ruling out the presence of high-risk varices in cirrhotic patients. *Digestive Diseases and Sciences*.

[32] Kim T. Y., Kim T. Y., Kim Y., Lim S., Jeong W. K., Sohn J. H. (2016). Diagnostic performance of shear wave elastography for predicting esophageal varices in patients with compensated liver cirrhosis. *Journal of Ultrasound in Medicine*.

[33] Sharma P., Kirnake V., Tyagi P. (2013). Spleen stiffness in patients with cirrhosis in predicting esophageal varices. *American Journal of Gastroenterology*.

[34] Fierbinteanu-Braticevici C., Tribus L., Peagu R. (2019). Spleen stiffness as predictor of esophageal varices in cirrhosis of different etiologies. *Scientific Reports*.

[35] Grgurević I., Bokun T., Mustapić S. (2015). Real-time two-dimensional shear wave ultrasound elastography of the liver is a reliable predictor of clinical outcomes and the presence of esophageal varices in patients with compensated liver cirrhosis. *Croatian Medical Journal*.

[36] Berzigotti A. (2017). Non-invasive evaluation of portal hypertension using ultrasound elastography. *Journal of Hepatology*.

[37] Qi X., Li Y., Wang R. (2019). Liaoning score for prediction of esophageal varices in cirrhotic patients who had never undergone endoscopy: a multicenter cross-sectional study in liaoning province, China. *Advances in Therapy*.

[38] Augustin S., Pons M., Maurice J. B. (2017). Expanding the Baveno VI criteria for the screening of varices in patients with compensated advanced chronic liver disease. *Hepatology*.

[39] Wang H., Wen B., Chang X. (2021). Baveno VI criteria and spleen stiffness measurement rule out high-risk varices in virally suppressed HBV-related cirrhosis. *Journal of Hepatology*.

[40] Cho Y. S., Kim Y., Sohn J. H. (2020). Application of supersonic shear imaging to the Baveno VI criteria and a combination model with spleen stiffness measurement to rule out high-risk varices in compensated advanced chronic liver disease. *Ultraschall in der Medizin-European Journal of Ultrasound*.

[41] Colecchia A., Ravaioli F., Marasco G. (2018). A combined model based on spleen stiffness measurement and Baveno VI criteria to rule out high-risk varices in advanced chronic liver disease. *Journal of Hepatology*.

